# New Paleocene Sepiid Coleoids (Cephalopoda) from Egypt: Evolutionary Significance and Origin of the Sepiid ‘Rostrum’

**DOI:** 10.1371/journal.pone.0081180

**Published:** 2013-11-21

**Authors:** Martin Košťák, John W. M. Jagt, Robert P. Speijer, Peter Stassen, Etienne Steurbaut

**Affiliations:** 1 Institute of Geology and Palaeontology, Faculty of Science, Charles University, Prague, Czech Republic; 2 Natuurhistorisch Museum Maastricht, Maastricht, The Netherlands; 3 Katholieke Universiteit Leuven, Leuven, Belgium; 4 Royal Belgian Institute of Natural Sciences, Brussels, Belgium; Australian Museum, Australia

## Abstract

New coleoid cephalopods, assignable to the order Sepiida, are recorded from the Selandian/Thanetian boundary interval (Middle to Upper Paleocene transition, *c*. 59.2 Ma) along the southeastern margin (Toshka Lakes) of the Western Desert in Egypt. The two genera recognised, *Aegyptosaepia* n. gen. and ?*Anomalosaepia* Weaver and Ciampaglio, are placed in the families Belosaepiidae and ?Anomalosaepiidae, respectively. They constitute the oldest record to date of sepiids with a ‘rostrum-like’ prong. In addition, a third, generically and specifically indeterminate coleoid is represented by a single rostrum-like find. The taxonomic assignment of the material is based on apical parts (as preserved), i.e., guard, apical prong (or ‘rostrum-like’ structure), phragmocone and (remains of) protoconch, plus shell mineralogy. We here confirm the shell of early sepiids to have been bimineralic, i.e., composed of both calcite and aragonite. *Aegyptosaepia lugeri* n. gen., n. sp. reveals some similarities to later species of *Belosaepia*, in particular the possession of a distinct prong. General features of the phragmocone and protoconch of the new form are similar to both *Belocurta* (Middle Danian [Lower Paleocene]) and *Belosaepia* (Eocene). However, breviconic coiling and the presence of a longer ventral conotheca indicate closer ties with late Maastrichtian–Middle Danian *Ceratisepia*. In this respect, *Aegyptosaepia* n. gen. constitutes a link between *Ceratisepia* and the Eocene *Belosaepia*. The occurrence of the new genus near the Selandian/Thanetian boundary suggests an earlier origin of belosaepiids, during the early to Middle Paleocene. These earliest known belosaepiids may have originated in the Tethyan Realm. From northeast Africa, they subsequently spread to western India, the Arabian Plate and, probably via the Mediterranean region, to Europe and North America.

## Introduction

Coleoid cephalopods of Paleocene age are very rare; their scarce fossil record currently includes a single stem-lineage sepiid group, represented by two genera, *Ceratisepia* and *Belocurta* [1,2]. The former probably is the earliest known sepiid, the type species, *C*. *elongata*, being from Danian (lower Paleocene) deposits at Vigny, Val-d’Oise (north-central France) [1]. Subsequently, a late Maastrichtian species from the southeast Netherlands, *Ceratisepia vanknippenbergi*, has been recorded [3]. This form co-occurs with the belemnitellid coleoids *Belemnitella junior* and *Belemnitella lwowensis* [4–6]. The genus *Belocurta* has been recorded form the ‘Montian’ (= Middle Danian [7]) of Israel and southern Belgium [1,2,8]. Both *Ceratisepia* and *Belocurta* precede the diverse and relatively common belosaepiids (family Belosaepiidae) during the Eocene [9], in particular the genera *Belosaepia* [10] and *Hungarosaepia* [11], as well as anomalosaepiids and mississaepiids [26,29]. *Belosaepia* and the younger *Hungarosaepia* are generally considered to form the stem lineage of the order Sepiida [12], i.e., to be ancestral to the Recent *Sepia* [13]. Eocene sepiids have been studied in detail by numerous authors [10,14–29]; all belosaepiids of the genus *Belosaepia* are confined to strata of Eocene (Ypresian–Bartonian) age. 

Another belosaepiid genus, *Anomalosaepia*, was erected to accommodate material of Ypresian to Bartonian age from North Carolina (USA [30]). A second North American form, *Mississaepia* [26], is of Bartonian to Priabonian age. Subsequently, *Anomalosaepia* has been assigned to its own family, the Anomalosaepiidae [28], while *Mississaepia* has lately been placed in a new family as well, the Mississaepiidae [29]. 

Here we present new and stratigraphically well-calibrated sepiid records from the Middle/Upper Paleocene (Selandian/Thanetian boundary interval) of southern Egypt. A new genus and species, *Aegyptosaepia lugeri* n. gen, n. sp., and incompletely preserved remains of ?*Anomalosaepia* represent the earliest ’rostrum-bearing‘ belosaepiids and anomalosaepiids known to date. Unlike *Ceratisaepia* and *Belocurta*, which do not possess a distinct prong (i.e., a ‘rostrum-like’structure) in the apical part, *Aegyptosaepia* n. gen. does have a well-developed prong. We consider the origin of the prong to be linked to the secretion of differently oriented crystal-forming layers (see below). A single specimen in the present collection, which represents an oval-rounded prong(?), does not permit any precise assignment within the Coleoidea. However, it is markedly different from all specimens of *Aegyptosaepia* n. gen. and ?*Anomalosaepia* in the same samples; it probably represents a third Paleocene coleoid taxon in southern Egypt.

## Methods

Specimen numbers: IGP-SL 09-7; IGP-BDA 09/01-10; IGP-BDB 09/1-6 stored at the collections of the Chlupáč´s Museum of Earth History (Institute of Geology and Palaeontology), Faculty of Science, Charles University in Prague (see below). 

All necessary permits were obtained for the present study, which complied with all relevant regulations. They were provided by the South Valley University, Qena (Dr M. Youssef). All samples were taken in the non-protected area, do not represent any commercial potential, and are not reported in any lists (CITES, etc.). They were legally exported to Belgium based on agreement between the KU Leuven and the South Valley University, Qena (Dr M. Youssef). We thank Dr Mohamed Youssef for logistic support. 

### Geological Setting and Stratigraphy

**Figure 1:**
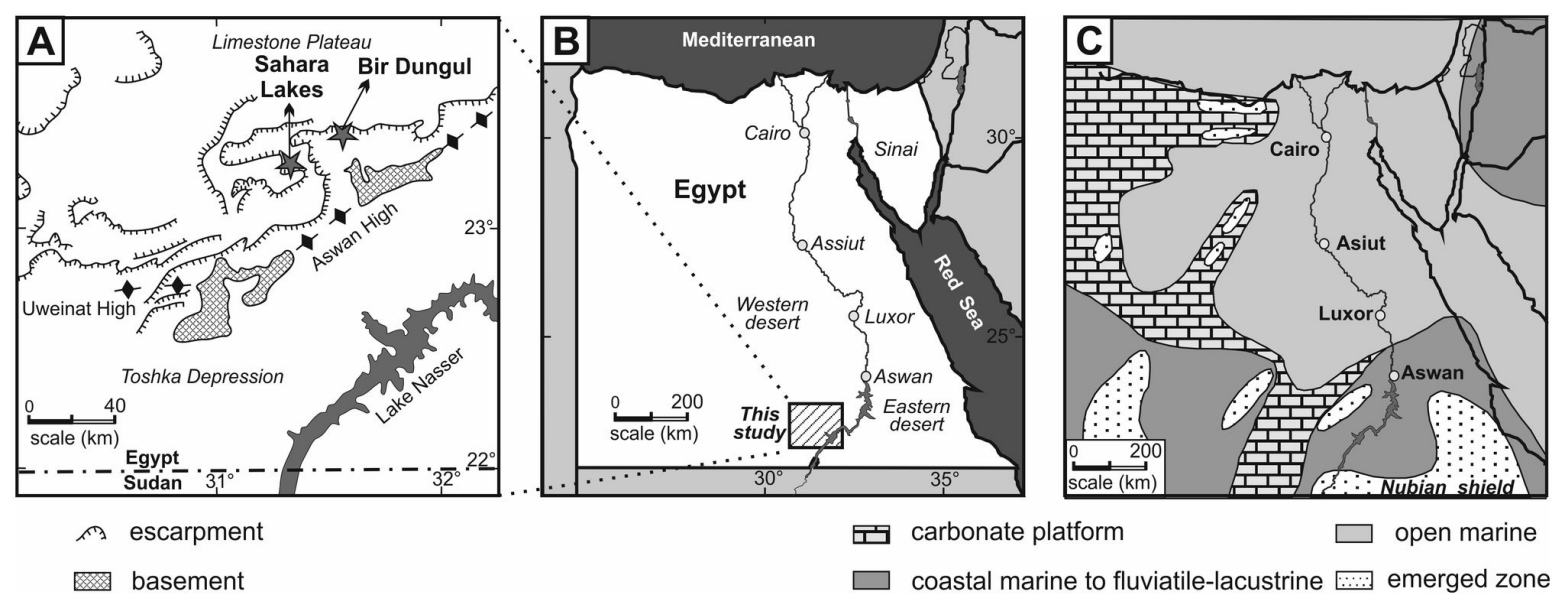
Geographical and palaeogeographical maps of Egypt. (A) Detailed location of the outcrops studied (modified from [66]). (B) Map of Egypt with study area indicated. (C) Palaeogeographical reconstructions of the late Paleocene (modified from [67]). Fm = formation.

**Figure 2:**
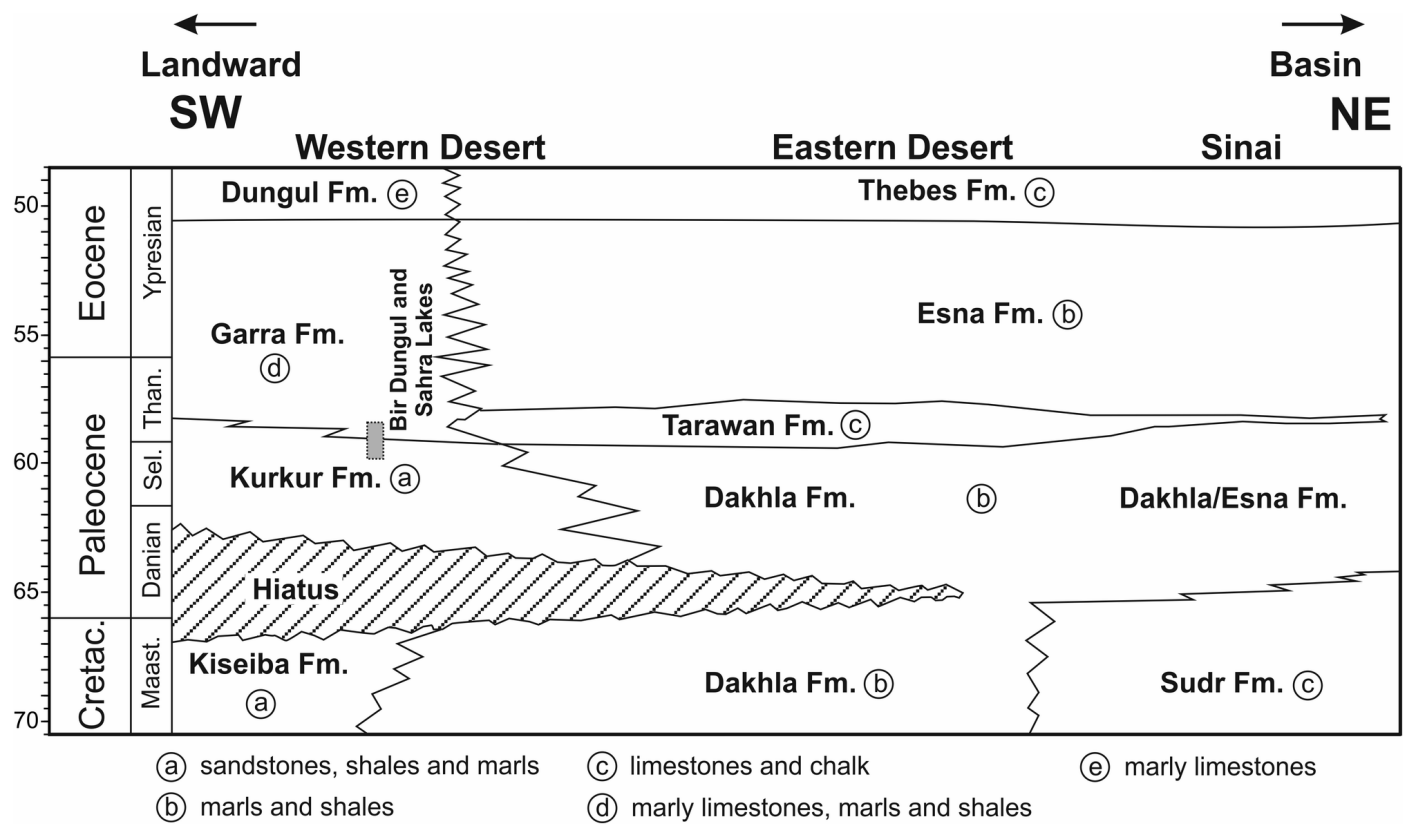
Schematic lithostratigraphical framework of the Maastrichtian to Ypresian interval in a northeast-southwest transect across Egypt (modified from [68]). The stratigraphical ranges of the outcrops studied are also indicated.

All available material comes from sites exposing Paleocene strata along the southeasterly edge of the Western Desert, west of the Aswan Reservoir, southern Egypt (Figure 1A-B), all apical parts (‘rostra’, prongs) were recovered from the Selandian/Thanetian boundary interval in the lower part of the Garra Formation (Figure 2). The three sepiid-bearing sites are as follows: (1) SL09-7 (‘Sahara Lake’ sample 2009-7), formed predominantly by chalky marl (Figure 1C), just along the east-west running road north of the most easterly Toshka Lake, an overflow lake from Lake Nasser (co-ordinates: 23N17’33.6”/31E20’32.3”; see Figure 1A). The strata probably correspond to the lower part of the Garra Formation. A single specimen of *Aegyptosaepia lugeri* n. gen., n. sp. was collected. Stratigraphically, the range of sediments outcropping corresponds to planktic foraminifera zones P4 (planktic foraminiferal Zone) or P5 (typical specimens of *Globanomalina pseudomenardii*, the marker of Zone P4 being usually missing in Egypt) and calcareous nannofossil zone NP7 (calcareous nannoplankton Zone), suggesting lower Thanetian; (2) Bir Dungul (section A) (=BDA) on the southern fringe of the limestone plateau (co-ordinates: 23N24’25”/31E37’24”; see Figure 1). This section represents part of the Garra Formation, formed by marls grading upwards into chalky marls and marly limestones. About 12 metres of section were sampled for micropalaeontological analysis (calcareous nannofossil analysis based on 12 samples); ten sepiids were collected from the surface of the outcrop. The stratigraphic position corresponds to planktic foraminiferal zones P3b–P4 at the base to P4-P5 at the top. The calcareous nannofossils indicate the upper part Zone NP5 at the base to Zone NP7 at the top, implying upper Selandian to lower Thanetian; (3) Bir Dungul (section B) (=BDB) on the southern fringe of the limestone plateau (co-ordinates: 23N24’55”/31E36’24”; see Figure 1). The Garra Formation exposed comprises marls that grade upwards into chalky marls and marly limestones. About 10 metres of section were sampled (nannofossil analysis based on 6 samples); six sepiid specimens have been recorded. Planktic foraminifera recorded are typical of zones P3b–P4 at the base to P3b–P5 at the top. Calcareous nannofossil zones range from top NP6 up to an undifferentiated interval upper NP7–lower NP8 (see below), indicating an early Thanetian age for this section.

The calcareous nannofossil associations from the Bir Dungul sections and Toshka Lake are fairly rich,well preserved (except for those from BDB, which are moderately to strongly dissolved) and moderately to highly diverse, indicating normal salinity sea surface waters. 

NP Zones [31] have been identified and subdivided on the basis of taxa recognised in coeval strata of the Aquitaine Basin [32] and at ODP Site 1262 (Walvis Ridge, southern Atlantic, 1,300 km west of the Namibian coast [33]. Following these data, BDA covers the upper part of Zone NP5 (lowest 1.50 m), Zone NP6 (2–4.50 m, marked by the presence of the NP5/NP6 boundary marker *Heliolithus kleinpellii* and the absence of the NP6/NP7 boundary marker *Discoaster mohleri*), the lower part of Zone NP 7 (5–6 m, marked by the co-occurrence of *D. mohleri* and *H. kleinpellii*) and an undifferentiated upper interval covering the upper part of Zone NP7 and the lower part of Zone NP8 (7–11 m). The latter is based on the presence of *D. mohleri*, the absence of *Heliolithus riedelii* (the NP7/NP8 boundary marker is to be expected here, but is extremely rare to absent at low latitudes, including Egypt) and the absence of the upper NP8 markers *Discoaster nobilis*, *Ericsonia universa* and *Discoaster okadai*, and of the NP8/NP9 boundary marker *Discoaster multiradiatus*, which are all known from other sites in Egypt [34].

The LO (lowest occurrence) of *Discoaster mohleri* is recorded at 0.5 m above the base of section BDB, indicating its position at *c.* 4.5 m above the base of DBA. The uppermost sample of BDB (9.60 m), is still within the undifferentiated interval including upper NP7–lower NP8. 

SL09-7 is characterised by the co-occurrence of *Heliolithus kleinpellii* and *Discoaster mohleri*, indicating the lower part of NP7, and a position slightly above the middle of BDA (within interval 5–6 m).

According to some authors [33], the base of magnetochron C26n, recently designated to define the base of the Thanetian Stage [35], lies between the lowest occurrences of *D. bramlettei* (at ~3.00 m in DBA) and *F. clinatus* (at ~4.00 m in DBA). This leads us to conclude that the lower 3.5 m of the BDA section is late Selandian in age and that the upper part of BDA, the entire BDB and sample SL09-7 are of early Thanetian age.

Based on calibration with the calcareous nannofossil biostratigraphy, the Bir Dungul sections appear to cover Subzone P3b up to probably the upper parts of Zone P4 [36]. The benthic foraminiferal assemblages from sections BDA, BDB and sample SL09-7 closely resemble those found at Gebel Duwi, near the Red Sea [37,38] and are of the Midway type, indicating middle to outer shelf conditions (palaeodepth 50–100 m; possibly deeper at certain levels in BDA) during deposition of the marls and chalks of the Garra Formation (Figure 2). 

### Material and specimen preparation

The material studied comprises 17 specimens (see below), of which more than half have been split and used for microscopic analysis. All specimens are housed in the collections of the Chlupáč´s Museum of Earth History (Institute of Geology and Palaeontology, Faculty of Science, Charles University) at Prague. SEM analyses have revealed strong recrystallisation; this has also been documented by other methods. Thus, the sepiid shells have been examined with the use of an optical microscope (Olympus SZX 12) and a mineralogical/petrological microscope (Nikon Eclipse, LV 100 Pol), using crossed nicols. Several shells, longitudinally cut and polished, have been assessed in thin sections. Some specimens have been coated by ammonium chloride prior to photography and cutting. Mineral identification is based on X-ray diffraction analysis (X´Pert Pro, PANalytical B.V. Almelo, at the Institute of Geochemistry, Mineralogy and Mineral Resources, Faculty of Science, Charles University in Prague).

### Terminology

**Figure 3:**
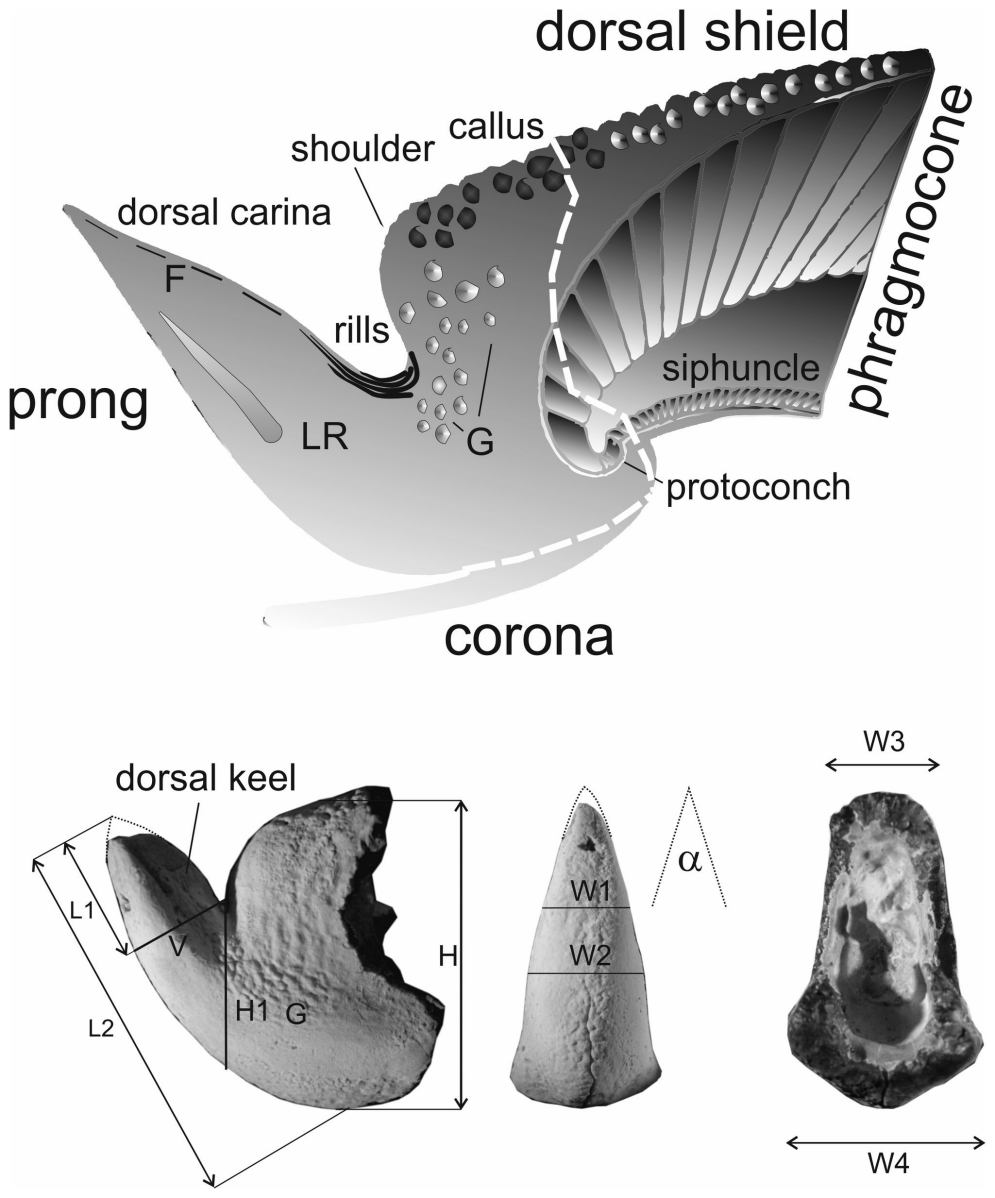
Terminology and dimensions measured in the apical part of belosaepiids (modified after [39]). Abbreviations: F – fissure; LR – lateral rib; G – granulation; L1 – length of prong; L2 – length from apex to base of corona; V – height of prong at junction with shoulder; H1 – height of prong between base of shoulder and ventral portion of prong in life position; H – height of guard; W1 – maximum width of free prong; W2 – maximum width of prong; W3 – dorsal width of guard; W4 – ventral width of guard; α – apical angle of prong.

**Figure 4:**
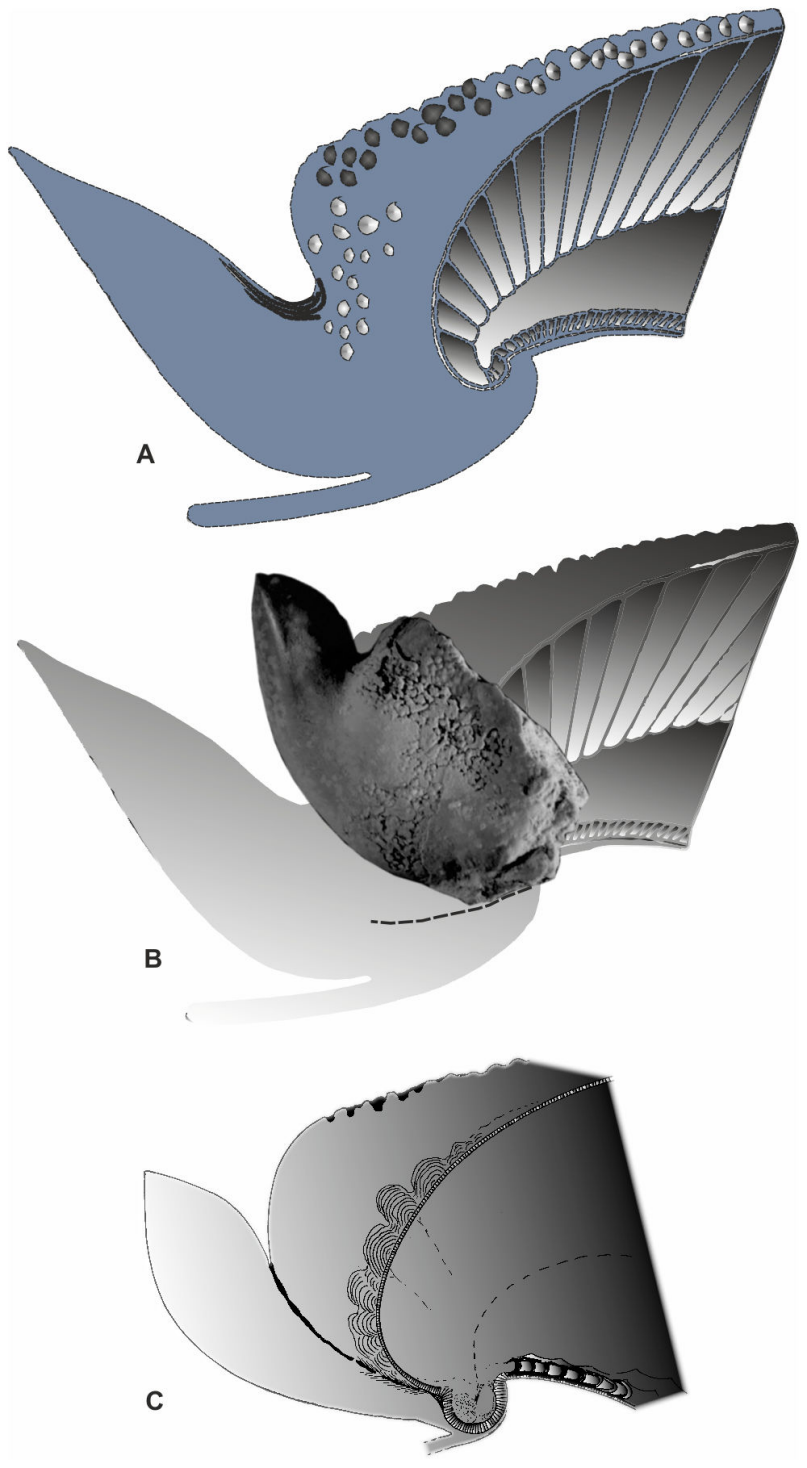
*Aegyptosaepia lugeri* n. gen, n. sp. (A) *Belosaepia* (drawing modified after [39]). (B) Paratype I (IGP-BDA 09/02), shown in life position, illustrating the different position of the prong in comparison with *Belosaepia*. (C) *Aegyptosaepia lugeri* n. sp. reconstruction based on thin sections studied.

Descriptive terms used in the present paper (Figure 3) are adopted from previous authors [27,39,40]. We prefer to use the term ‘rostrum-like structure’ or prong for the thorn-like protuberance (apical spine) at the posterior end (Figures 3, 4). The granular layer covering the dorsal part of the phragmocone, i.e., the sheath [39,40], should be replaced by the term ‘guard’ [27]. The term sheath should be restricted to the thick aragonitic layer that covers the conotheca at the dorsal apical part of the phragmocone (= sheath or primordial rostrum [41]; see discussion below). All other terms used herein correspond to those employed for Eocene belosaepiids from Texas [27]. A new term, ‘connecting fissure’ is introduced.

### Nomenclatural acts

The electronic edition of this article conforms to the requirements of the amended International Code of Zoological Nomenclature, and hence the new names contained herein are available under that Code from the electronic edition of this article. This published work and the nomenclatural acts it contains have been registered in ZooBank, the online registration system for the ICZN. The ZooBank LSIDs (Life Science Identifiers) can be resolved and the associated information viewed through any standard web browser by appending the LSID to the prefix "http://zoobank.org/". The LSID for this publication is: urn:lsid:zoobank.org:pub: 7D2A29CE-4BDA-41D3-83D4-437CB1696E0F. The electronic edition of this work was published in a journal with an ISSN, and has been archived and is available from the following digital repositories: PubMed Central, LOCKSS. 

## Results

### Systematic palaeontology

Coleoidea Bather, 1888 [42]

Sepiida Gray, 1849 [12] 

Belosaepiidae Dixon, 1850[9] 

*Aegyptosaepia* n. gen. 

#### Derivatio nominis

After Egypt (Latin Aegyptus), where the genus has been recorded for the first time. The second part, -*saepia*, indicates relationship to the order Sepiida.

#### Diagnosis

Relatively small (guard height ~ 15 mm), heavily mineralised sepiid with well-developed, short and robust, asymmetrically lanceolate prong; guard quadrangular in cross section; rounded shoulder with granulation on either side; strongly mineralised callus with tubercles (nodes, bumps); base of corona relatively far removed from apex of prong; prong inclined dorsally; breviconic phragmocone endogastrically coiled and with ventral wall (as in *Ceratisepia*) formed by conotheca; shell composed of both calcite (guard and prong) and aragonite (sheath covering dorsal apical part of phragmocone).

#### Description

The shell is strongly mineralised, including a short, well-developed prong (~ 7 mm). The prong is shaped like a knife with a sharp dorsal margin, the cross section being triangular. The prong markedly inclines dorsally. The shoulder and majority of the callus show tubercles on the dorsal side. The corona is quite distant from the base of free prong. The breviconic phragmocone is endogastrically coiled, the spherical protoconch large, *c.* 1 mm in diameter. Caecum probably present. 

#### Discussion

*Aegyptosaepia* n. gen. differs from the early Paleocene guard-bearing sepiid *Belocurta* in having a well-developed, clearly distinguished prong, and from Eocene belosaepiids by the character of the prong (i.e., short and robust, dorsally markedly inclined and triangular in cross section), as well as by the large distance between the corona and the apex. Although the guard is more massive and robust, *Aegyptosaepia* n. gen. ranks amongst the smaller sepiids. According to the general characters of the protoconch and phragmocone, the new genus represents a transition between *Belocurta* and *Belosaepia*. However, some morphological features, such as the ventral phragmocone wall, show similarities to *Ceratisepia*.

*Aegyptosaepia lugeri* n. sp. 

urn:lsid:zoobank.org:act:E435B0C5-8BCD-4277-B0D5-283B5C644941

#### Types

**Figure 5:**
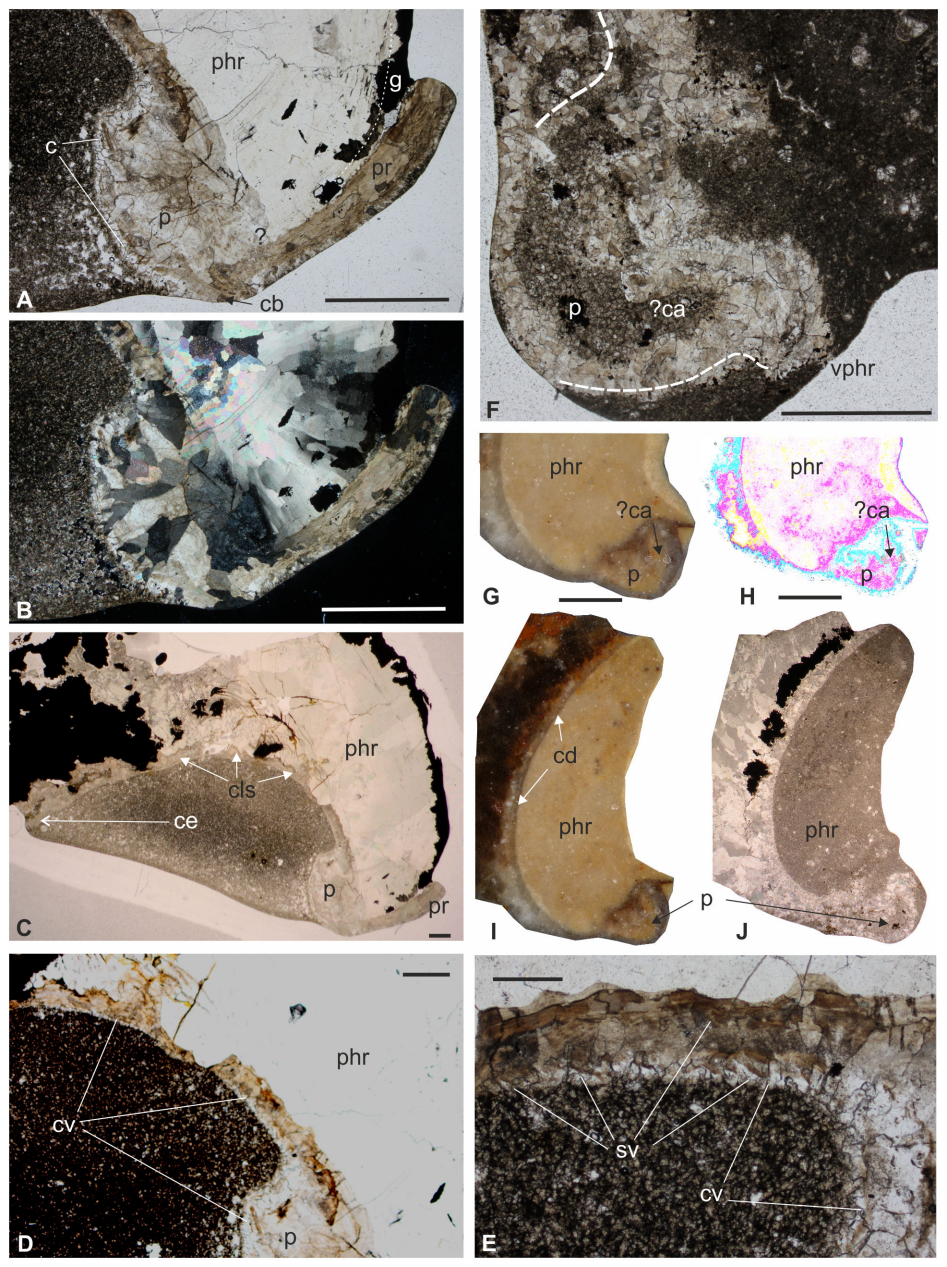
Thin section of compressed and fragmentary protoconch (A) of specimen IGP-BDA 09/04 (the part of the shell figured corresponds to the red rectangle in Figure B) (B) crossed nicols view, showing strong recrystallisation by calcite. Scale bar equals 1 mm. (C) Thin section of phragmocone of specimen IGP-BDA 09/04, with no dorsal septa preserved. Scale bar equals 2 mm. (D) Thin section of detail of ventral part of phragmocone with conotheca in specimen IGP-BDA 09/04. Scale bar equals 2 mm. (E) Thin section of detail of ventral part of phragmocone in specimen IGP-BDA 09/04, with remains of ventral septa preserved; septa partially attached to conotheca. Scale bar equals 0.5 mm. (F) Thin section of non-compressed protoconch, probably possessing caecum, in specimen IGP-BDA 09/07; dashed line follows shape of protoconch. Scale bar equals 1 mm. (G–H) Polished section of specimen IGP-BDA 09/07, apical part of phragmocone with position of protoconch. (H) view in trassed colours (Corel Photopaint X3 programme used). Scale bar equals 1 mm. (I–J) Polished and crossed-nicols thin section, respectively, of specimen IGP-BDA 09/07, phragmocone with protoconch. Scale bar equals 1 mm. Abbreviations: p – protoconch; phr – phragmocone; c – conotheca; cb – base of corona; g – guard; pr – prong; cls – *Ceratisepia*-like structures (?conellae); ce – place of conotheca disappearing; cv – conotheca at ventral part of phragmocone; ?ca – caecum; cd – conotheca at dorsal part of phragmocone; sv – remains of ventral septa; vphr – ventral part of phragmocone.

**Figure 6:**
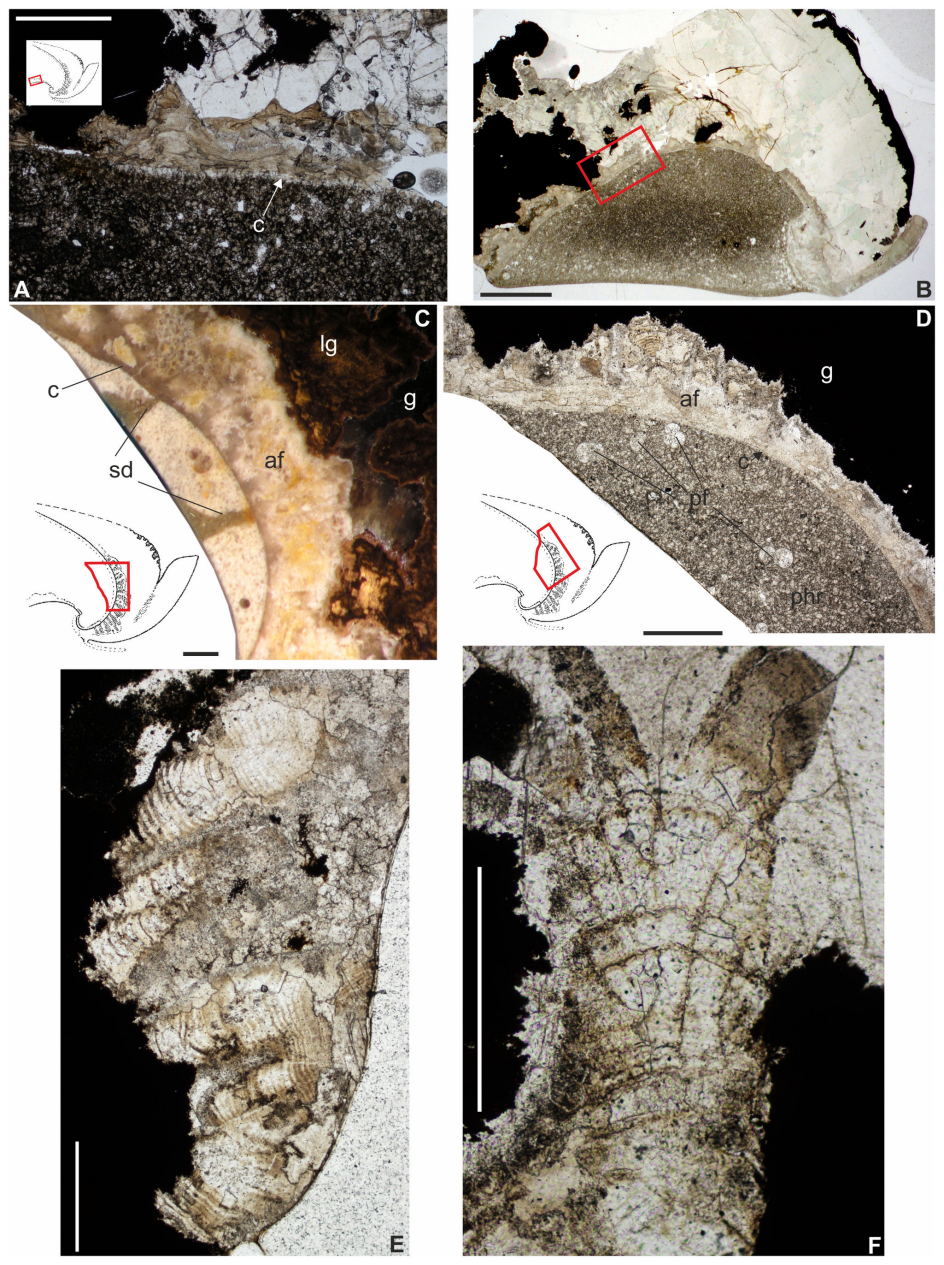
Thin section (A-B) illustrating *Ceratisepia*-like structures at ventral part of phragmocone (?conellae) in specimen IGP-BDA 09/04. Scale bars equal 0.3 mm (A) and 1 mm (B). (C) Polished section of specimen IGP-BDA 09/05, showing dorsal part of phragmocone with preserved remains of dorsal septa, aragonite fans (primordial rostrum) and calcitic (partially limonitised) guard (rostrum proper). Scale bar equals 0.5 mm. (D) Thin section of specimen IGP-BDB 09/01, illustrating alveolus infilled by sediment, bordered by conotheca, aragonite fans (primordial rostrum) and calcitic guard (rostrum proper). Scale bar equals 1 mm. (E–F) Thin section of specimen IGP-BDB 09/01, showing details of aragonite fans forming sheath (primordial rostrum). Scale bar equals 1 mm. Abbreviations: c – conotheca; phr – phragmocone; g – guard; sd – remains of dorsal septa; af – primordial rostrum (sheath), aragonite fans; lg – calcitic, partly limonitised guard; pf – planktic foraminifera.

**Figure 7:**
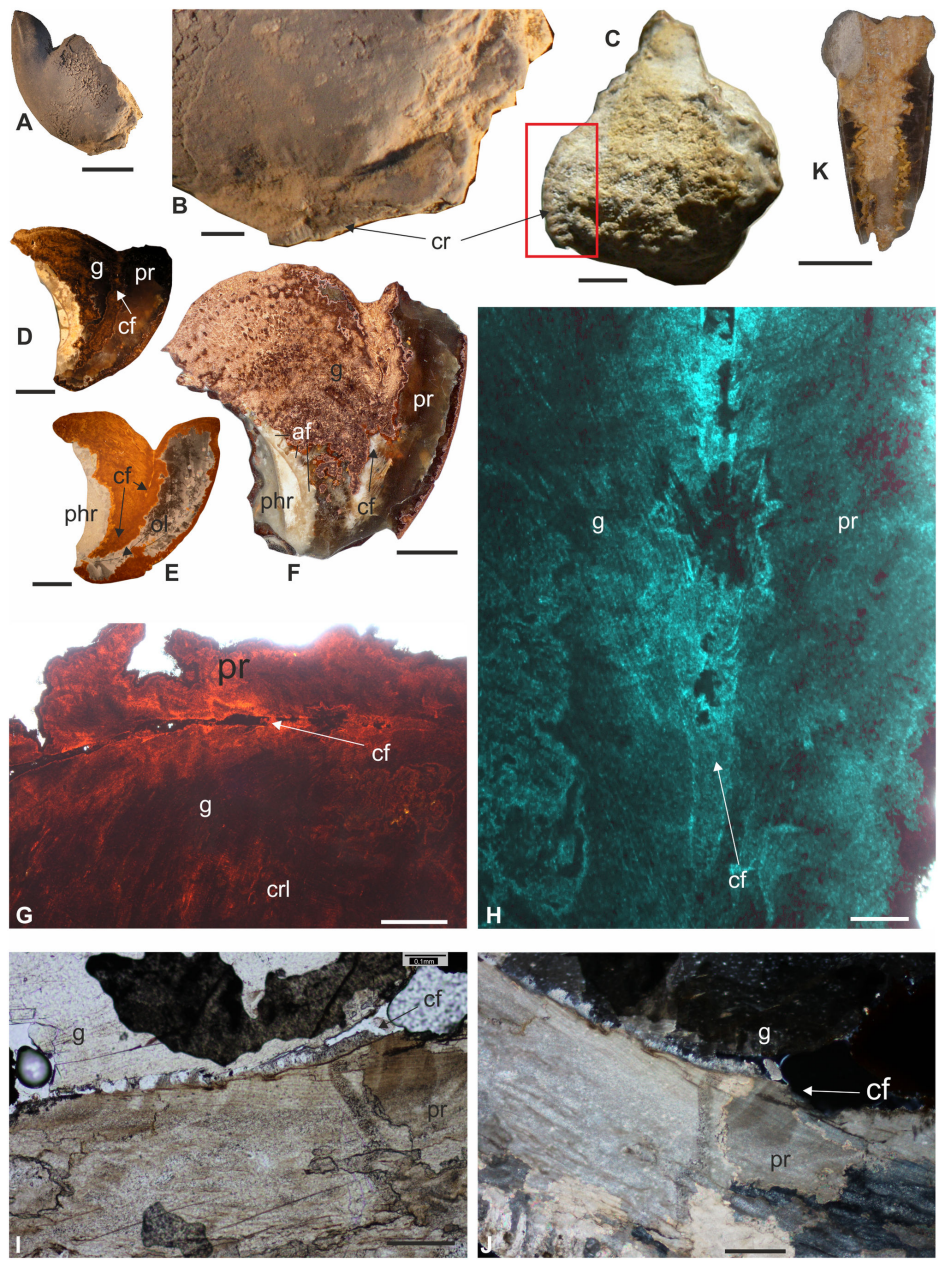
Paratype III (IGP-SL-07) with well-preserved base of corona. (A) Scale bar equals 10 mm. (B) Detail of base of corona. Scale bar equals 1 mm. (C) Specimen IGP-BDB 09/03, with preserved part of corona with radial ribs. Scale bar equals 5 mm. (D) Polished section of specimen IGP-BDA 09/05, showing position of connecting fissure. Scale bar equals 5 mm. (E) Thin section of Paratype II (IGP-BDB 09/01), with position of connecting fissure. Scale bar equals 5 mm. (F) Polished section of specimen IGP-BDB 09/02, with preserved part of alveolus, aragonitic sheath and partially limonitised calcitic guard. Scale bar equals 3 mm. (G) Connecting fissure between guard and prong, with elongated cavities, in Paratype II (IGP-BDB 09/01). Guard formed by calcitic concentric-radial layers. Scale bar equals 0.5 mm. (H) Detail of connecting fissure in Paratype II (IGP-BDB 09/01). Scale bar equals 0.1 mm. (G–H) Thin sections. (I–J) Beginning of connecting fissure in close proximity (10 mm) of protoconch; connecting fissure secondarily infilled by smaller ?carbonate crystals (J). Specimen IGP-BDA 09/04. Scale bar equals 0.1 mm. (I-J) – thin sections. (K) Straight and elongate ‘rostrum’ of unknown coleoid cephalopod, showing mineralisation features similar to those in *Aegyptosaepia* n. gen. Specimen IGP-BDA 09/06. Polished section. Scale bar equals 10 mm. Abbreviations: g – guard; pr – prong; cf – connecting fissure; phr – phragmocone; cr – corona rim remains; af – primordial rostrum (sheath), aragonite fans; ol – prong axial line; crl - concentric-radial layers.

**Figure 8:**
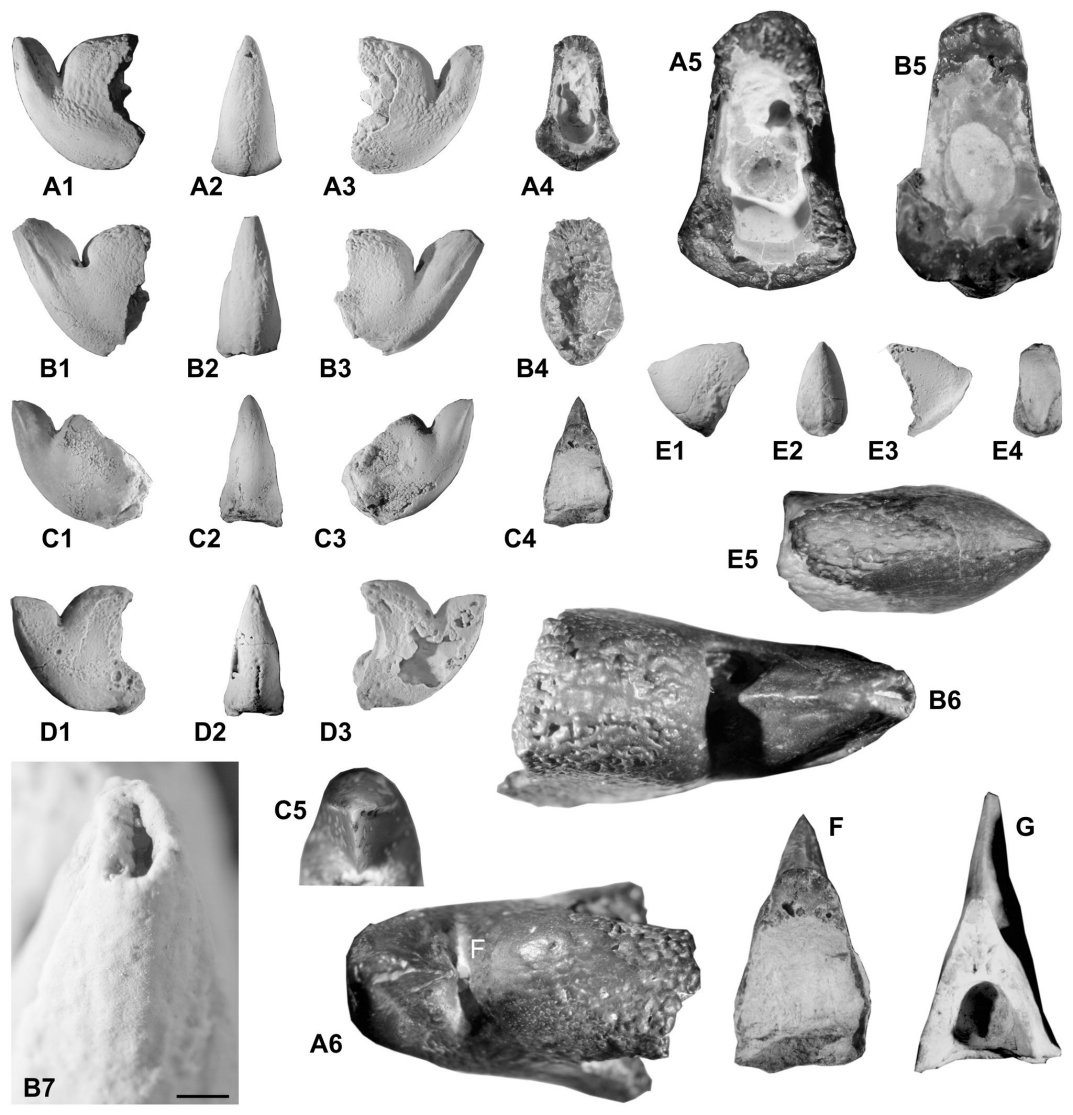
*Aegyptosaepia lugeri* n. gen., n. sp., holotype (IGP-BDA 09/01) in lateral (A1, A3) and apical (A2) views, in cross sections (A4-A5; A5 x 3) and dorsal view (A6), showing knife-like character of prong, shoulder and callus with tubercles developed. B. *Aegyptosaepia lugeri* n. gen., sp., Paratype I (IGP-BDA 09/02), in lateral (B1, B3) and apical (B2) views, in cross section (B5, x 3). B5 illustrates cross section of oval-rounded phragmocone, while B6 represents a dorsal view, showing knife-like prong, shoulder and callus with tubercles developed. Apex of prong with developed ventro-dorsal fissure, x 4.5. B7 shows detail of dorso-ventral fissure, x 5. C. *Aegyptosaepia lugeri* n. gen. n. sp., Paratype III (IGP-SL 09-7), in lateral (C1, C3) and apical (C2) views; C4 illustrates cross section at base of prong, while C5 illustrates detail of knife-like character of prong. D. *Aegyptosaepia lugeri* n. gen., n. sp., Paratype II (IGP-BDB 09/01), in lateral (D1, D3) and apical (D2) views. E. ?*Anomalosaepia* ( IGP-BDA 09/08) in lateral (E1, E3) and apical (E2) views, while E4 illustrates cross section and E5 dorsal view with tubercles developed, x 3.5. F–G. Comparison of cross section at protoconch between *Aegyptosaepia lugeri* n. gen., n. sp., Paratype III (IGP-SL 09-7) from southern Egypt and *Belosaepia* sp. (IGP 2011/C1) from southern Turkey, x 3.

The holotype is IGP-BDA 09/01; paratypes are IGP-BDA 09/02, IGP-BDB 09/01 and IGP-SL-07. Dimensions of type specimens are listed in Table 1. Eight specimens with measurable parameters and a few strongly abraded and uncertain fragments; four from Bir Dungul section A, three from Bir Dungul section B and a single specimen (SL09-7) from along the east-west trending road north of the most easterly Toshka Lake, an overflow lake of Lake Nasser (Figures 4–7, 8A–D, F).

**Table 1:** Dimensions (in mm) of specimens studied.

Reg. no.	L1	L2	H	H1	W1	W2	W3	W4	V	α	gran
IGP-BDA 09/01 **holotype**	7.6*	21.7	16.8	8.2	6.2	8.1	10.3	5.9	4.9	32	x
IGP-BDA 09/02 **Paratype I**	8.2*	20*	15.1	8.9	4.9	6	8.5	6.2	6.1	31	x
IGP-BDB 09/01 **Paratype II**	5.8	20	15	7.4	6.3	8.4			5.8	32	
IGP-SL 09-7 **Paratype III**	6.4	20.8	16*		4.7	6.3	9.8	5.4	5.2	36	
IGP-BDA 09/03 juv.			12.2	6.2		4.8	7	4.9	3.5		x
IGP-BDB 09/02	8.6			9.8	6.3	8.4	6.1	11.7	7.1	31	
**average**	**~7**	**~21**	**~15**	**~8.1**	**~5.7**	**~7**	**~8.3**	**~6.8**	**~5.4**	**~32.4**	

* estimated.

#### Etymology

Named after Dr Peter Luger (Technische Universität Berlin), whose thorough stratigraphical and micropalaeontological PhD research in southern Egypt [43] initiated our study of the Paleocene-Eocene Garra Formation in the area.

#### Locality and horizon

The holotype and Paratype IGP-BDA 09/02 are both from the southeasterly margin of the Western Desert, Bir Dungul section A, co-ordinates 23N24’25” /31E37’24” (Figure 1), the southern fringe of the limestone plateau. Paratype IGP-BDB 09/01 is from the same area, Bir Dungul section B, co-ordinates 23N24’55”/31E36’24” (Figure 1), the southern fringe of the limestone plateau. Lower part of the Garra Formation [43], upper Selandian to lower Thanetian (Middle to Upper Paleocene, ~59.2 Ma [44]); calcareous nannoplankton zones NP5–NP7; planktic foraminiferal zones P3–P4.

#### Description

Comparatively small sepiid with well-mineralised posterior portion (guard) consisting of prong and callus. Average values: length L2 ~ 21 mm; width W1 ~ 5.5 mm; width W2 ~ 7 mm; W3 ~ 8 mm; W4 ~ 7 mm; height H ~ 15 mm), inclusive of the solid prong (Figures 3, 7E, 8A1–A3, B7). Prong apparently short (L1 ~ 7. 5 mm) and robust, asymmetrically lanceolate (i.e., knife-like) in lateral aspect and conical (α ~ 32°) in dorso-ventral aspect. Prong with well-developed sharp carinal margin (Figure 8A6, B6, F) in dorsal part. Prong nearly straight on ventral side, dorsal side showing marked curvature formed by knife-like edge. Visible extension situated at one-third of posterior part of prong. Cross section of prong strictly triangular, inclining dorsally at 60–70° from horizontal axis. Dorsal and ventral edges diverging at approximately 55°. Both dorsal and ventral surface of prong almost smooth, possessing fine granulation in posterior part in holotype and Paratype IGP-BDA 09/02. No striation developed, not even in rill field area (Figure 8A6). Fissure visible at apex (Figure 8A1, B1, C1). Marked conical lateral ribs taper towards apex, passing into dorsolateral planes/depressions towards dorsal carina. 

Shoulder and most of callus with tubercles on dorsal sides (Figure 8A6, B6), probably indicating earlier ontogenetic stage [compare 27–28]). Majority of callus/dorsal shield not preserved; shoulder distinct in holotype and Paratype IGP-BDA 09/02; Figure 8A-B). Ventral corona not completely preserved, but its base retained as coronal rim on posteroventral surface of guard (Figure 7A–C) and/or as flexure line. Of great importance is position of base of corona within guard (Figure 7A), which is further removed from apex of prong in comparison to later belosaepiids.

In specimens IGP-BDB 09/01, IGP-BDA 09/05 and IGP-BDB 09/02 (Figure 5D–F), the nature of origination of the prong is probably illustrated (see below). A thin layer, here named (new term) ‘connecting fissure’ (Figure 7D–J), divides the majority of the guard from the new structure, the prong. In thin sections and polished sections (Figure 7G-H), different crystal orientations have been observed. The interface layer (?an originally organic membrane and/or the place of the mantle tissue, respectively) starts closely to the protoconch area (Figure 7I-J). Some smaller cavities, of irregular development, occur around the connecting fissure. 

#### Phragmocone and protoconch

The phragmocone is incompletely preserved and only a few morphological features are visible. One of the most relevant macroscopic morphological features is the coiling of the phragmocone. In comparatively better-preserved specimens, i.e., IGP-BDA 09/4 and IGP-BDA 09/7, most of the apical part of the phragmocone, inclusive of the protoconch part, is preserved (Figure 5). Phragmocone coiling corresponds to that of *Belocurta* and *Belosaepia*. No complete dorsal septa are preserved, with the exception of their attachments parts (Figure 6C) in the dorsal mural part of the phragmocone. The ventral septa are well preserved in specimen IGP-BDA 09/4 (Figure 5E), in which they form a strong planar deck structure [27]. The thickness of the deck amounts to 0.3–0.4 mm. In this area, septa are recurved and attached to the conotheca at an obtuse angle. The ventral wall of the phragmocone is relatively long, comparable to the situation seen in *Ceratisepia* (Figure 5C-D). However, these conical structures (compare [1]; see Figure 6A-B herein) have been observed in thin section only; these might represent conellae, well known in other extinct coleoid cephalopods, in particular belemnites. The thickness of the conotheca increases (*c.* 1.5 mm from the protoconch: 0.1–0.2 mm) towards the protoconch (0.4 mm, at the protoconch). The ventral phragmocone wall (conotheca) continuously effaces from the protoconch towards the anterior part. Approximately 4–5 mm from the protoconch, the conotheca fuses into a very thin layer (?membrane) and continues posteriorly in an irregular manner. From this point onwards, the ventral phragmocone wall does not follow phragmocone coiling. The siphuncle band has well-developed, typically undulate margins. The bases of the ventral septa appear to be covered by a thin layer (Figure 5E), possibly equating with the ?lamello-fibrillar nacre (*sensu* [29]). However, a very thin layer also covers their strongly recurved dorsal parts (?connecting strips sensu [45]). Ventral septa are visible only in a 4-mm-long segment adhering to the protoconch.

The protoconch is relatively large, hemispherical, almost 1 mm long and 1.5 mm wide, with a prismatic shell wall. In specimen IGP-BDA 09/4, the protoconch area is heavily compressed, including numerous fragments of the phragmocone and protoconch walls (Figure 5A). In this case, the phragmocone is secondarily filled and mineralised by calcite crystals (Figure 5B). Specimen IGP-BDA 09/7 shows the original shape of the protoconch and, probably, the position of the caecum (Figure 5F–H). A closing membrane cannot be documented in view of the state of preservation. 

### Shell Mineralisation

**Figure 9:**
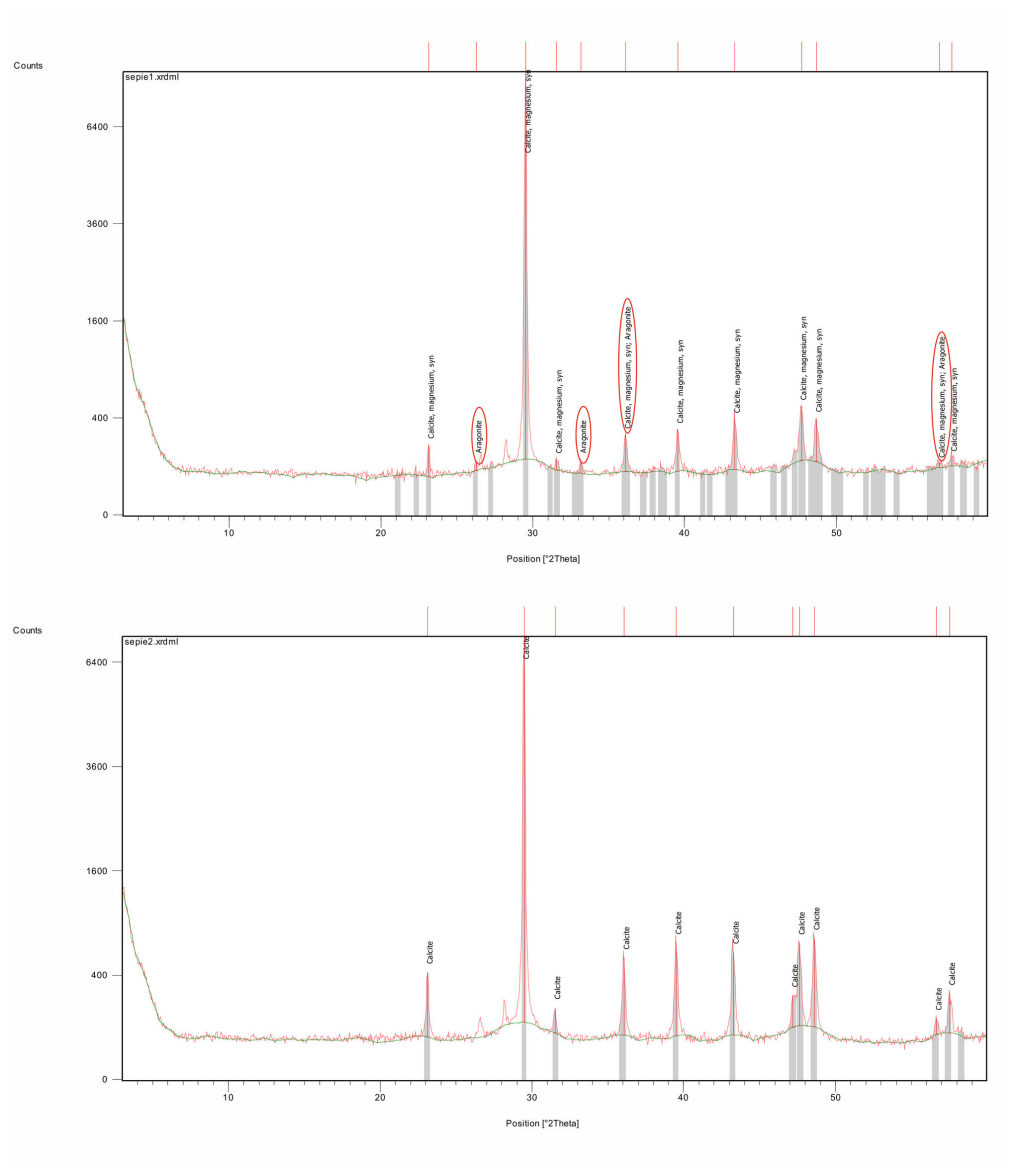
Mineralogical composition of the sheath, showing original presence of aragonite (A), recorded calcite also contains a Mg-element. (B) Mineralogical composition of the prong showing pure calcite. X-ray diffraction (X´Pert Pro, PANalytical B.V., Almelo).

The shell of the Recent cuttlefish *Sepia* is composed of principally inorganic (predominantly aragonite and some calcite and hydroxyapatite) and some organic (β-chitin and proteins) matter [45]. A similar composition is seen in extinct representatives of the *Sepia* stem lineage, in particular in *Ceratisepia*, *Belocurta*, *Belosaepia* and *Anomalosaepia*. Recently, extremely well-preserved cuttlebones of *Mississaepia* have been recorded [29,46] that revealed a similar inorganic-organic sepiid cuttlebone composition. However, the sepiid shells described herein are strongly recrystallised (Figures 5–6). Yet, some important microstructural data are preserved thanks to mineral pseudomorphosis (substitution), clearly documenting the original mineral composition and the original composition has been detected using RTG (X-ray diffraction ) methods. X-ray diffraction (Figure 9) has shown the original composition of the guard to have been bimineralic, i.e., composed of both calcite and aragonite (Figures 6C–F, 7D–F), a feature which is also well visible in crystal differentation (i.e. Figures 6E-F, 7G-H). Our analysis has revealed that the first layer covering the conotheca is composed of aragonite (however, due to strong recrystallisation, Mg-calcite forms a dominant, yet secondary component). It forms radial fans, which have also been recorded in the Eocene *Anomalosaepia* [28]. In this respect, this layer should be considered the equivalent of the primordial rostrum of belemnoids (see below). The second layer, forming the guard and prong, are composed of poor calcite, with no traces of aragonite and magnesium. The phragmocone is poorly preserved and only a few parts of the protoconch and ventral part of the siphonal tube are preserved as semi-transparent, brownish to yellowish remains located closely to the mural parts. Probably, originally less mineralised septa (?chitinous) are not preserved. 

Secondarily, the posterior parts of the shells were strongly recrystallised by calcite (Figure 6C-D), except in some parts. The final, post-diagenetic, phase of mineralisation is suggested here to have been limonitisation (Figures 6C, 7D–F), invading in particular calcitic portions (but, in part, preserving the original morphology, including microstructures) of the guard and prong. Although the fossilisation process appears quite chequered and recrystallisation led to damage of certain morphological structures, the most relevant data are preserved.

### Discussion and Remarks

The problem of the ‘rostrum’ in coleoid terminology has recently been analysed in admirable detail [41]; the granular layer, rostrum-like structure in sepiids was suggested to represent a secondary or tertiary formation. According to [41], the sepiid sheath (= guard herein) corresponded to the belemnite primordial rostrum, because sepiids possessed only a single outer formation outside the conotheca. In a redescription [28] of the genus *Anomalosaepia*, it was shown that the guard (sheath/primordial rostrum, respectively) was composed of radial-prismatic and fan-radial aragonite and the outer layer of non-prismatic calcite crystals. In this respect, the outer calcitic part should be considered to be the equivalent of the ‘rostrum proper’ (*sensu* [41]). Additionally, a thin basal prismatic layer covering the conotheca and below the spherulitic-prismatic layer has been recorded in *Mississaepia* [29]; a similar layer arrangement has been observed in specimens decribed herein. The basal prismatic layer covering the conotheca is formed by aragonite and it is irregularly developed in the apical part in *Aegyptosaepia* n. gen. (Figures 6C–F, 7D–F). A similar shell arrangement has also been observed in the Late Cretaceous belemnite family Belemnitellidae [47], in particular in the genera *Praeactinocamax* [48,49] and *Goniocamax* [48,50]. In the former, the apical part of the phragmocone is surrounded by aragonitic layers, while in *Goniocamax* this space is considered to have been originally organic and formed by more or less mineralised ‘capsulae’ [50]. However, the possible presence of a caecum in *Aegyptosaepia* n. gen. (see above) differentiates the earliest sepiids from belemnoid coleoids. 

We here present a new interpretation of the origin of the ‘rostrum-like structure’ in early sepiids, i.e., belosaepiids. On the basis of the thin sections studied, the prong consists of the same structures (lamellae in combination with radial structures) as the guard, but it is strictly separated from the prong and crystal orientations are opposing. Previous authors [27] have documented a well-defined narrow transition zone between the prong and the guard. The ‘connecting fissure’ (new term) represents a strict boundary between the prong and the guard. It is formed by a thin layer which starts closely to the protoconch area. The fissure is secondarily and irregularly infilled by needle-like crystals. Some smaller cavities are observable. We suggest that the fissure is connected to a mantle tissue fold, secreting countercurrent crystals. A similar idea was introduced for the weak fissure in *Belosaepia* by previous authors [27]; however, this fissure is perpendicular to the connecting fissure. 

According to [27], the thickness of the septa is 0.1 mm or less in some belosaepiids; they are structurally weak and lost *post-mortem*. However, it is possible that septa were less mineralised and/or composed of organic matter (?chitin; see 29). The bases of the ventral septa correspond to ‘leur racine (pse)’ as illustrated by [1]. This phenomenon has recently been reinterpreted by [27], who considered the presence of a very thin conotheca covered ventrally by a thicker endoventral prismatic layer [29]. noted a very thin lamello-fibrillar nacre layer, covering probably organic mural parts of septa in the genus *Mississaepia*. However, the structure of this part of the phragmocone is complex and not yet fully understood [29].

Anomalosaepiidae Yancey & Garvie, 2011[28]
?*Anomalosaepia* Weaver & Ciampaglio, 2003 [30]

#### Material

A single specimen, IGP-BDA 09/08, comprising the well-preserved apical part of the guard (Bir Dungul section B), and two uncertain fragments (Bir Dungul section A) (Figure 8E1-5), Selandian–Thanetian boundary interval, Garra Formation, calcareous nannofossil Zones NP5–NP7, planktic foraminiferal Zones P3–P4.

#### Description

Sepiid with well-mineralised posterior part of the shell. The guard is 6.4 mm wide and 12.7 mm high. The prong is very small (2 mm long, 1.7 mm wide and 3.5 mm high), triangular in lateral view and oval in cross section, inclining dorsally at 32–35° from the horizontal axis. Dorsal and ventral edges diverge at approximately 75°. The ventral side of the guard possesses a slightly developed ventral, keel-like structure (Figure 8E2). The cross section of the guard is oval-rectangular, laterally flattened. The ventral part is wider (W3 = 6.4 mm) than the dorsal (W4 = 5 mm). Neither fissure, nor striation are visible. 

The shoulder and most of the callus show tubercles at the dorsal sides (Figure 8E5), probably indicating earlier ontogenetic stages (compare [27]). The majority of the callus is not preserved, nor is the ventral corona. 

#### Discussion

**Figure 10:**
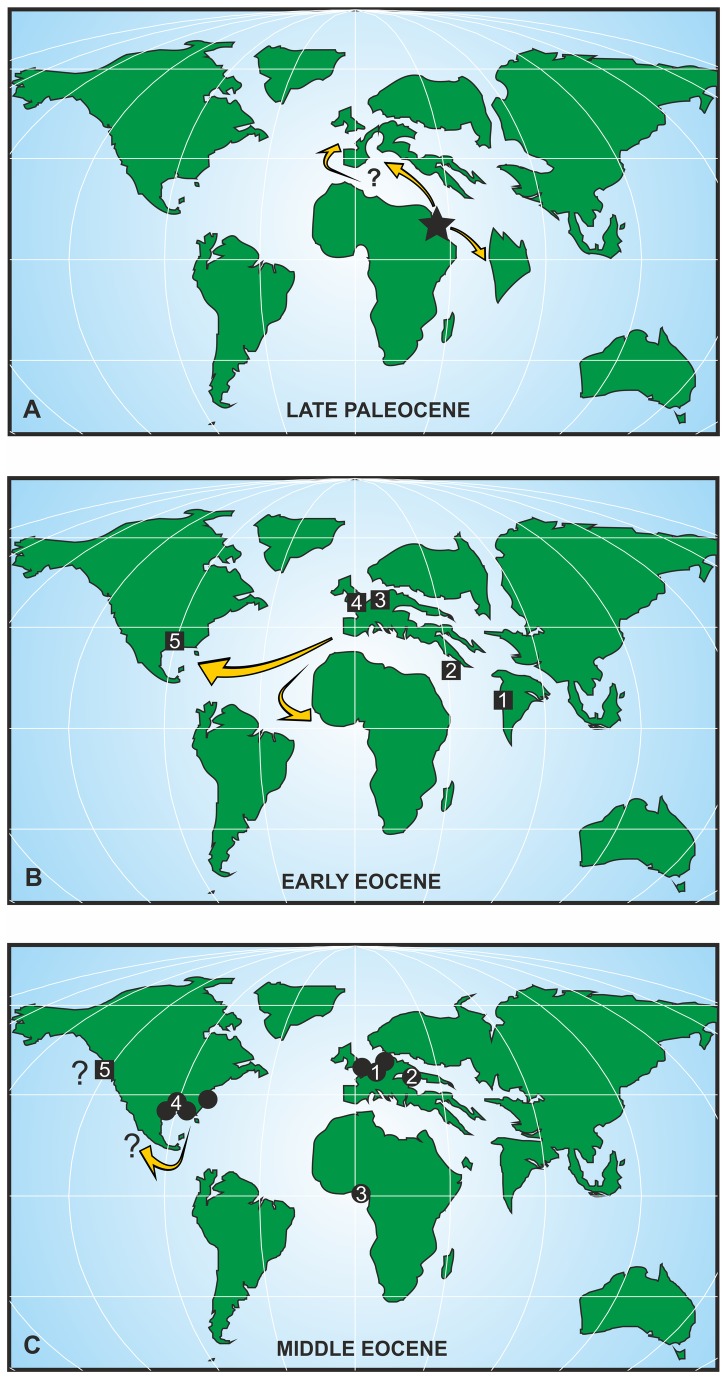
Palaeogeographical distribution of belosaepiids s. lat. (inclusive of the genus *Hungarosaepia* and the anomalosaepiid *Anomalosaepia*). (A) The first belosaepiid occurrence in the Middle/Late Paleocene (Selandian–Thanetian) in northeast Africa (asterisk: *Aegyptosaepia lugeri* n. gen., n. sp., Egypt) and possible migratory routes. (B) Palaeobiogeographical distribution of belosaepiids during the Early Eocene. 1. *Belosaepia incurvata*, Sind, western India (Early Eocene, Ypresian). 2. *Belosaepia* sp., southeast Turkey (Early Eocene, Ypresian). 3. *Belosaepia* ex gr. *sepioidea*, Belgium (Early Eocene, Ypresian). 4. *Belosaepia* ex gr. *sepiidea*, England (Early Eocene, Ypresian). 5. *Belosaepia* s. str., USA (Texas; Early Eocene, Ypresian). (C) Palaeobiogeographical distribution of belosaepiids during the Middle Eocene. 1. *Belosaepia* ex gr. *sepioidea*, France, England, Belgium, the Netherlands (Middle Eocene, Lutetian–Bartonian). 2. *Belosaepia szoerenyii*, Hungary (Middle Eocene, Lutetian). 3. *Belosaepia sepioidea*, southern Nigeria (Middle Eocene, Lutetian). 4. *Belosaepia* s. str., USA (Alabama, Louisiana , Texas), North Carolina (*Anomalosaepia*) (Middle Eocene, Lutetian–Bartonian). 5. Occurrence of ?belosaepiid in the Late Eocene, USA (northwest Washington). * Yellow arrows denote possible migratory routes. Palaeogeography corresponds to the Late Paleocene (Thanetian), Early to Middle Eocene. Maps have been based, schematised and modified from www.scotese.com and http//cpgeosystems.com (Ron Blakey).

These specimens resemble the Eocene genus *Anomalosaepia*, which is known exclusively from North America [26,28] (Figure 10C). However, only the posterior portion is retained; despite this, the general morphological features, such as the prong and callus, correspond to this genus. Although anomalosaepiids, of which four species have been described to date (in which only the posterior part is known), resemble belosaepiids, a new family, Anomalosaepiidae [28], has been established on the basis of a unique mineralogy (i.e., both calcite and aragonite), microstructures and unusual guard morphology. 

Another enigmatic coleoid, *Oligosella* [51], from the Oligocene of Alabama (USA), shows some general morphological similarities to our specimens, in particular in the lateral shape of the guard. However, this genus is poorly known and should be considered embryonic [26,51]. In addition, *Oligosella* is stratigraphically much younger than ?*Anomalosaepia* as described herein. 

?*Anomalosaepia* vaguely resembles some strongly abraded specimens of *Aegyptosaepia lugeri* n. gen., n. sp., but the shape and specific morphological features (characteristics of the guard and prong in ?*Anomalosaepia*), as well as the near-perfectly preserved surface in specimen IGP-BDA 09/08 clearly rules out their conspecificity. In view of the fact that only incomplete specimens are available to date, we refrain from establishing a new taxon. 

 Previous authors [28] suggested that *Anomalosaepia* was restricted to the Eocene of the western Atlantic region of the United States. We here present a morphologically very close coleoid taxon from older Paleocene deposits in southern Egypt. However, based on the rare and incomplete material, we are not in a position to confirm whether the North American anomalosaepiids are conspecific with *Anomalosaepia* described herein. Nevertheless, the Egyptian taxon clearly demonstrates a higher sepiid diversity during the Paleocene than previously reported.

Coleoidea *incertae sedis*
?Sepiida Gray, 1849 [12]

#### Material

A single specimen of a ‘rostrum-like structure’ (?prong), IGP-SL09-10 (Figure 7K), from along the east-west trending road north of the most easterly Toshka Lake, an overflow lake from Lake Nasser.

#### Description

This free ‘rostrum-like structure’ consists of a well-rounded (oval) fragment, 17 mm in length, 6 mm in width and 7 mm in height. There are no growth lines, except for the last one. The middle part (axis) is recrystallised and secondarily infilled by different material (carbonate and limonite). The surface is smooth, no ribs are developed. The most apical part is broken. 

#### Discussion

The internal character of the ?prong is closely similar to prongs of *Aegyptosaepia lugeri* n. gen., n. sp. (Figure 8A–D). However, the length, shape and cross section differ markedly from all specimens of *A. lugeri* n. gen., n. sp. on record. The ?prong is 2–3 times longer. The oval cross section is markedly different from that of *Aegyptosaepia lugeri* n. gen., n. sp., which is triangular. Although the single specimen is poorly preserved, it probably represents a third coleoid taxon from the Selandian/Thanetian boundary interval in southern Egypt.

### Sepiid evolution during the Late Cretaceous to Eocene

Based on molecular studies, sepiids and spirulids are thought to have diverged near the Jurassic/Cretaceous boundary (~150 Ma +/- 30 myr) [52]. Previous authors [53] proposed an even earlier divergence time of sepiids in the Late Permian at 260 Ma. However, these divergence ages are not (yet) supported by the palaeontological record. The first record of Sepiida is at about 68–70 Ma, but these first (latest Cretaceous and Paleocene) sepiids are extremely rare; major phylogenetic features based on shell morphology clearly document an earlier evolution.

The earliest sepiid on record is the genus *Ceratisepia* [1] from the late Maastrichtian of southeast Netherlands (*C*. *vanknippenbergi* [3] and Danian of north-central France (*C. elongata* [1]). The relationship of *Ceratisepia* to Eocene belosaepiids is based on similarities of the breviconic phragmocone. However, some differences are noted in size, muscle attachment and, in particular, embryonic features, such as the more elongated aperture and lower and less endogastrically coiled median profile at the same width in *Belosaepia* [3]. One author [54] considered the late Maastrichtian *C*. *vanknippenbergi* to be congeneric with the Hauterivian–Turonian (Early to early Late Cretaceous) belemnoid (diplobelid) genus *Conoteuthis* [55], which was considered to be the earliest sepiid. However, a detailed study of new records of *Conoteuthis* with well-preserved phragmocones, inclusive of the protoconch, has led to the rejection of the sepiid nature of *Conoteuthis* [56].

The highly interesting sepiid *Belocurta* (with three species) from the Danian of Israel and southern Belgium lacks a ‘rostrum-like structure’ or prong. The guard simply surrounds the breviconic phragmocone and protoconch. The ‘procesum ventrale’ (= corona) of [1] is faintly developed in *Ceratisaepia*. In *Belocurta*, the corona is better developed, but its major portion is closely connected to the guard. The phragmocone is less endogastrically coiled, and especially the protoconch shows morphological features in common with later belosaepiids. 

According to our new records from the Paleocene of Egypt, the belosaepiid *Aegyptosaepia* n. gen. represents a transitional taxon between a *Belocurta-*like sepiid and *Belosaepia*. Phragmocone coiling in *Aegyptosaepia* n. gen., such as the spherical protoconch characteristics correspond already to the exclusively Eocene belosaepiids. A new evolutionary feature is the prong, which is first recorded in *Aegyptosaepia* n. gen. Of key importance is the presence of aragonitic ‘anomalosaepiid-like’ microstructures. The systematic value of bimineralic shells, however, remains to be assessed in greater detail. Still, it is questionable, if this morphological feature is of value in family-level assignment. 

**Figure 11:**
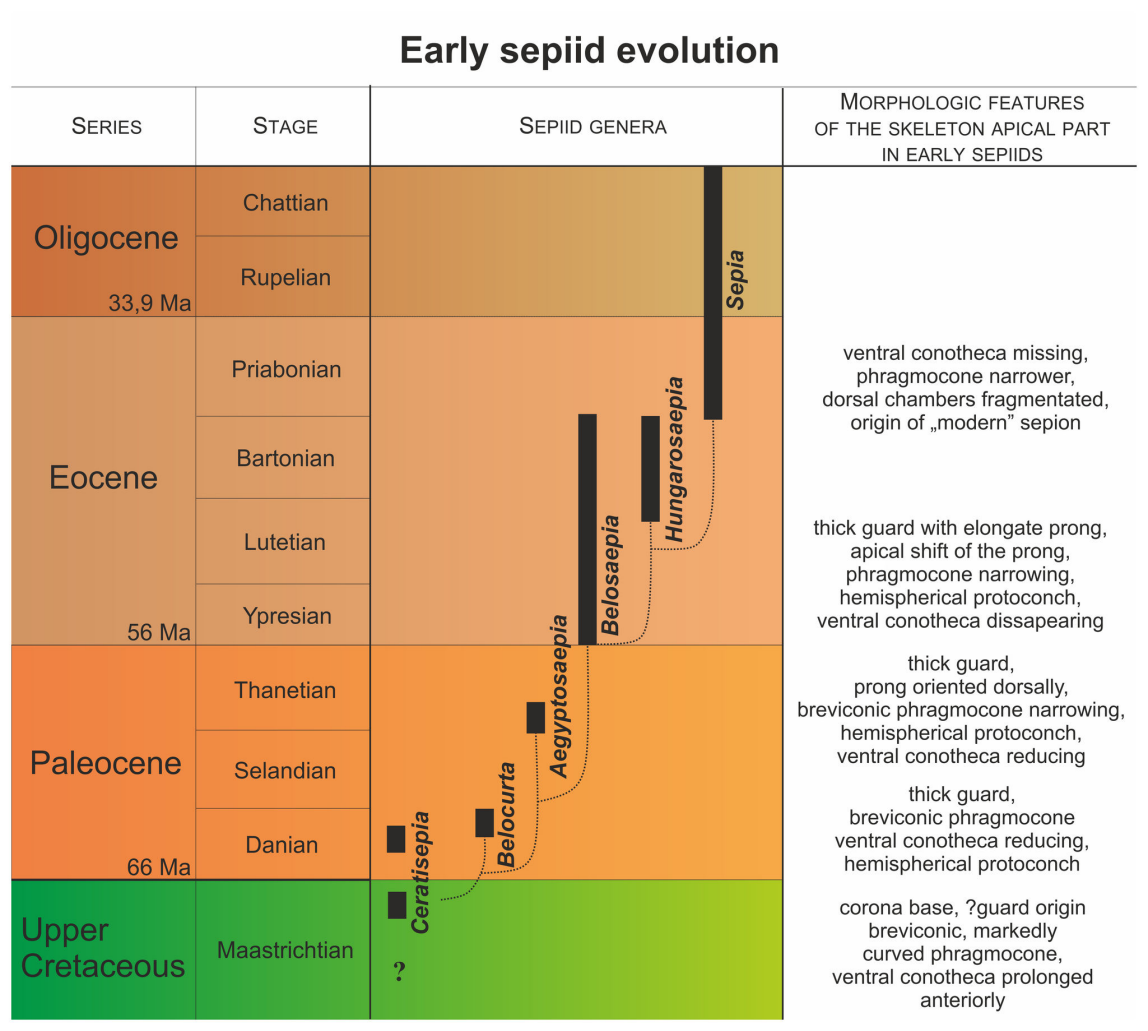
Stratigraphical distribution of the earliest sepiids, with possible phylogenetic relationships and major morphological features recorded.

Eocene sepiids (family Belosaepiidae, and, in particular the genus *Belosaepia*) are considered to be a stem-lineage of coleoids of the order Sepiida, i.e., ancestors of the Recent *Sepia*. Previous authors [3] noted the possibility of a phylogenetic transition between *Belosaepia* and *Sepia* via *Hungarosepia* (see also 11). In view of the fact that both *Ceratisepia* and *Belocurta* have been recorded from the lower Paleocene, we assume the divergence to have occurred around the Cretaceous-Paleogene (K/Pg) boundary (Figure 11). The genus *Belocurta* should be viewed as the ancestral candidate of *Aegyptosaepia* n. gen. for the following reasons: (1) similar shape and size of protoconch, (2) almost identical shape and coiling of the phragmocone in longitudinal section, and (3) similar characteristics of ventral septa. On the other hand, the cross section of the phragmocone and general characteristics of the guard and ?corona are different. Additionally, *Aegyptosaepia* n. gen. arose rather rapidly (~ 2.8 myr) after *Belocurta* and already reveals more progressive morphological features that are typical of *Belosaepia* and some microstructural (mineralogical) features typical of *Anomalosaepia* and *Mississaepia*. In this respect, *Aegyptosaepia* n. gen. probably represents a stem-group of belosaepiids s. lat. 

The hypothetical evolutionary patterns of early sepiids, based on changes in the morphology of the apical part (see also Figure 11), are as follows:

1. origin of the guard protecting the phragmocone; phragmocone coiled; *Ceratisepia-*type protoconch, i.e. the *Ceratisepia* phase;
2. increase in guard thickness; phragmocone straightening; hemispherical protoconch, i.e., the *Belocurta* phase;
3. origin of the prong, connected to mantle overlap and secretion of overgrowths covering the guard; prong markedly inclined dorsally; successive disappearance of the ventral phragmocone wall, i.e., the *Aegyptosaepia* n. gen. phase.
4. reduction of guard thickness; prong elongation and straightening; phragmocone modification, ventral phragmocone wall disappearing, i.e., the *Belosaepia* phase.

We assume that all of these these morphological changes were linked to buoyancy control.

### Palaeobiogeography and palaeoecology

Late Cretaceous to Eocene sepiids are confined to the Northern Hemisphere, with the exception of a Middle Eocene record from southern Nigeria [57]. The highest species diversity has been recorded from the lower to Middle Eocene of northwest Europe. The earliest ‘rostrum-bearing’ sepiids have been recorded from the lower Eocene (Ypresian) of northwest Europe (England, France, Belgium, Germany, the Netherlands), the southern USA (Texas) and the Tethyan Realm, e.g. India and southern Turkey [58]. However, Tethyan records are few and far between; previous authors [58] mentioned the possibility that belosaepiids might have originated in the Tethyan Realm. This idea is supported herein by our new records representing the earliest ‘rostrum-bearing’ belosaepiid known (i.e., Selandian/Thanetian boundary interval) from southern Egypt.

Based on the material described herein, we assume belosaepiids to have originated in the northwest Tethys (i.e., shelf seas of northeast Africa) during the Paleocene. In view of the fact that *Aegyptosaepia lugeri* n. gen., n. sp. shows more signs of ‘modern’ (i.e., Eocene) belosaepiids, the origin of the group may be suggested to have lain in the early Paleocene and/or near the K/Pg boundary. It is generally accepted that phragmocone-bearing Late Cretaceous coleoids (represented predominantly by belemnitellids) were missing from the Tethyan Realm, especially during the latest Cretaceous [59] with the exception of a rare record of the genus *Belemnitella* is that from the northern edge of the Arabian Plate, from a highly restricted stratigraphic interval, close to the Campanian/Maastrichtian boundary [60]. Thus, from the latest Cretaceous onwards, the northwest Tethys represented a region full of free niches. Probably from this area, the late Paleocene to early Eocene rostrum-bearing sepiids migrated to western India [61] and southern Turkey [60], and, most likely, through the Mediterranean area also to northwest Europe and via the central Atlantic Ocean as far west as southern North America (Figure 10).

The highest sepiid diversity (in particular belosaepiids and related genera) has been recorded from the Middle Eocene of Europe [19,21,25,62] and the southern USA [26,27]. Both areas have an extremely high number of species, and should be considered to be radiation centres. However, belosaepiids from Europe and USA are in need of a major taxonomic revision, because many of these taxa seem to be conspecific. Other records of Middle Eocene belosaepiids (*B*. *sepiidea*) are those from southern Nigeria [57] and probably Egypt [63]. However, the position of *B*. *arabica* [63] within the genus *Belosaepia* remains unclear, as does that of a late Eocene belosaepiid record from northwest Washington [64].

It should be noted that the stratigraphic distribution of early sepiids (s. str.) conspicuously coincides with global events. Their geographic distribution and the highest taxonomic diversity seem to be associated with palaeogeographic changes and, especially, with major climatic changes. ‘Rostrum’-bearing sepiids originated approximately with the start of the pronounced warming trend from the base of the late Paleocene at ~ 59.2 Ma [44]. Their widest geographic distribution occurred in the early Eocene (~ 56-49 Ma; i.e., Early Eocene Climatic Optimum) [65]; species persisted in cooler, albeit still high, temperature conditions in the Middle Eocene. The genus *Belosaepia* s. str. disappeared at the end of the Middle Eocene. The extinction may have been caused by a progressive, but significant cooling trend from the late Middle Eocene to the Eocene/Oligocene boundary terminated by rapid temperature fall, recorded also from the deep sea [65].

In general, belosaepiids are considered to have been benthic taxa [27]. According to sedimentological and micropalaeontological data, our new records from Egypt suggest a middle to outer shelf habitat. In the BDA section (marls grading upwards into chalky marls and marly limestones – similar to the BDB section), a Midway-type benthic foraminiferal assemblage (see above) with occasional deep shelf markers has been recorded associated with 40–70% planktic taxa suggesting a middle to outer shelf setting. The palaeodepth may be estimated to have been 50–200 m. The BDB section also yields a Midway-type benthic foraminiferal association and 60–80% planktic taxa, thus conditions showing middle shelf environment with depths of about 50–100 m. A single specimen recorded from the locality Sahara Lake is associated with a Midway-type benthic foraminiferal assemblage with 50% planktic taxa. It indicates again middle shelf palaeoenvironmental conditions with depths ranging between 50–100 m. The new records from Egypt show either unusual taphonomy and/or, more likely, their true habitat. In this respect, we consider the earliest prong-bearing sepiids to have been typical bottom dwellers, inhabiting a middle shelf sea floor (50–200 m). A benthic life habit is also indicated by the large protoconch [27]. Thus, the first representatives (and also probably their ancestors) were middle-to-deep shelf inhabitants (bottom dwellers), rather than occupants of shallower water/nearshore environment with higher dynamics. The same conditions are considered for the second and uncertain sepiid genus described herein, ?*Anomalosaepia* from sections BDA 09 and BDB 09. 

The prong origin in *Aegyptosaepia* n. gen. probably is connected with buoyancy control. Because of the dorsal inclination, the possibility of the prong functioning as a tool for burrowing should be rejected. The early ‘rostrum-bearing’ sepiids were bottom dwellers; thus, their origin is suggested to have been benthic. The taphonomic results (see above) strongly support this view.

## Conclusions

The new Paleocene (Thanetian/Selandian boundary) belosaepiid genus and new species, *Aegyptosaepia lugeri*, represents a taxon that links latest Cretaceous and Eocene sepiids. It possesses a mosaic of morphological features that connect late Maastrichtian *Ceratisepia* and Eocene *Belosaepia*, with the majority of ‘modern’ (i.e., belosaepiid and anomalosaepiid) characters. The presence of a hemispherical protoconch, probably with a caecum, differentiates the sepiid stem lineage from belemnoids. However, the presence of a sheath (= aragonitic primordial rostrum [41]) covered by a calcitic layer (= rostrum proper), shows a very close similarity to the Late Cretaceous belemnite family Belemnitellidae.

*Aegyptosaepia* n. gen. represents the earliest known taxon with a prong (= ‘rostrum-like structure’) that developed in the apical portion of the bimineralic shell. The origin of the prong is here proposed to be a result of calcite secretion by a folded mantle which responded to needs of buoyancy control (i.e., counterbalance). The mantle fold engraved the connecting fissure, an important morphological feature between the guard and the prong. The dorsal inclination of the prong clearly rejects previous theories of an original function in burrowing. 

*Aegyptosaepia lugeri* n. gen., n. sp. hints at a northwest Tethyan (North Africa) origin of prong-bearing sepiids, and its descendants probably spread to Europe and North America. Belosaepiid distribution is limited by the Early Eocene climatic optimum and higher temperature in the Middle Eocene. At least two other coleoids, albeit rare, are recorded herein from southern Egypt, which adds significantly to Paleocene coleoid cephalopod diversity. Based on foraminiferal data, the original habitat of *Aegyptosaepia* n. gen. is considered to have been a middle shelf sea floor (50–100 m, possibly up to 200 m): the large protoconch suggests it was a bottom dweller. 
